# Supplementary material for: Changes in Gut Microbiota According to Disease Severity in a Lupus Mouse Model
Source: Int J Mol Sci. 2025 Jan 24;26(3):1006. doi: 10.3390/ijms26031006 (PMC11817498; doi:10.3390/ijms26031006)
Supplement: Supplementary file 1 [file ijms-26-01006-s001.zip › ijms-3427716-supplementary.pdf]

Supplementary Materials

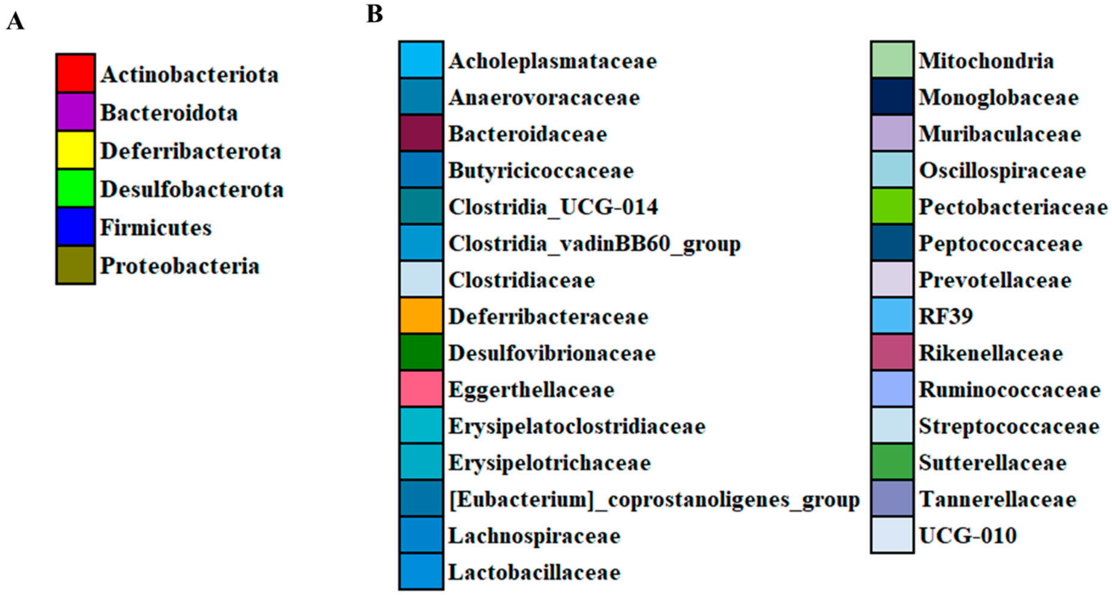

Figure S1. Composition of Fecal Microbiota. (A) Taxonomic classification of fecal microbiota a t the phylum level, represented by different colors. (B) Detailed classification at the family le vel, with each color corresponding to a specific bacterial family.

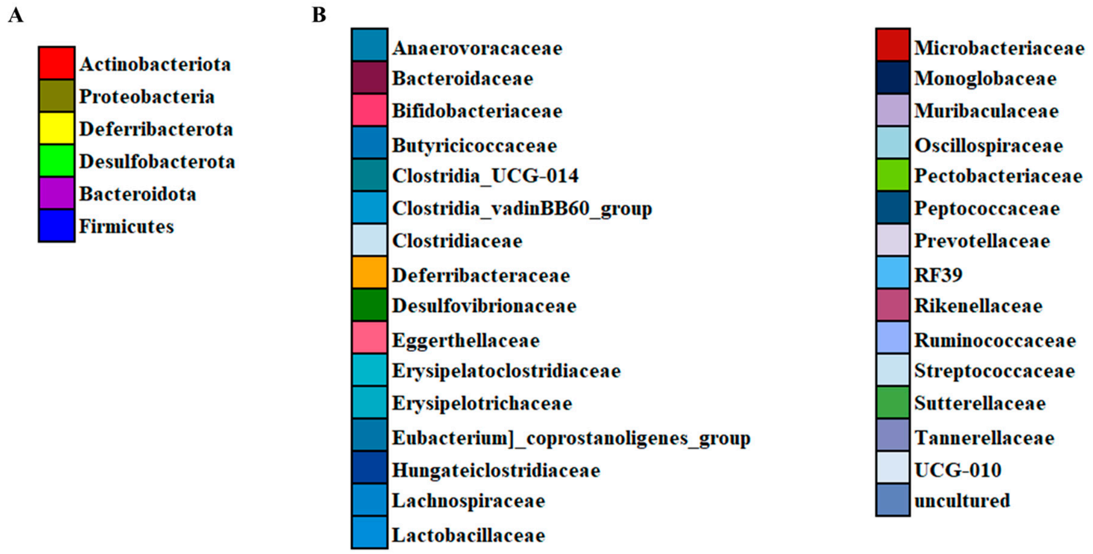

Figure S2. Composition of GI Microbiota. (A) Taxonomic classification of GI microbiota at the phylum level, represented by different colors. (B) Detailed classification at the family level, w ith each color corresponding to a specific bacterial family.

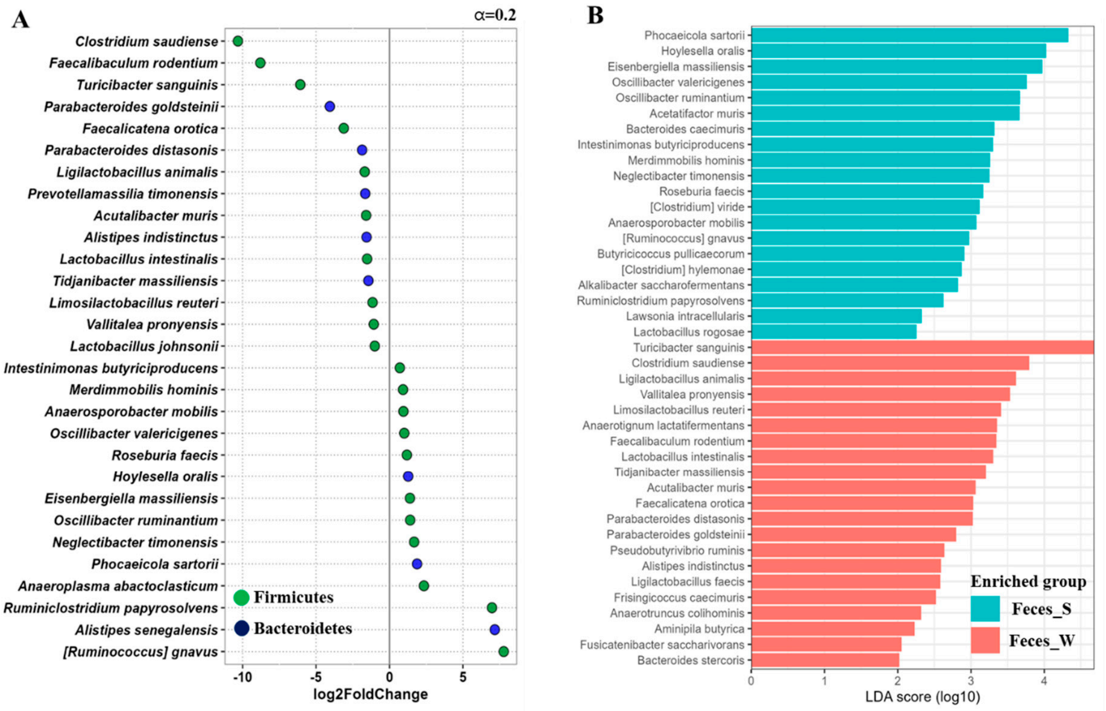

Figure S3. Differential analysis of fecal gut microbiota in wild-type (W) and severe lupus (S) MRL/lpr mice. (A) Volcano plot showing differentially abundant fecal microbial species between severe lupus (Feces_S) and wild-type (Feces_W) mice, as determined by DESeq analysis using read counts. Species from the phylum Firmicutes are shown in green, while species from Bacteroidetes are shown in blue. Positive values indicate species enriched in severe lupus mice, while negative values indicate those enriched in wild-type mice. (B) Linear discriminant analysis (LDA) effect size (LEfSe) plot identifying differentially enriched fecal microbial species between severe lupus (Feces_S) and wild-type (Feces_W) mice based on read counts. Species enriched in the severe group are shown in blue, while those enriched in the wild-type group are shown in red. Higher LDA scores indicate a greater contribution to the respective group's microbial composition.

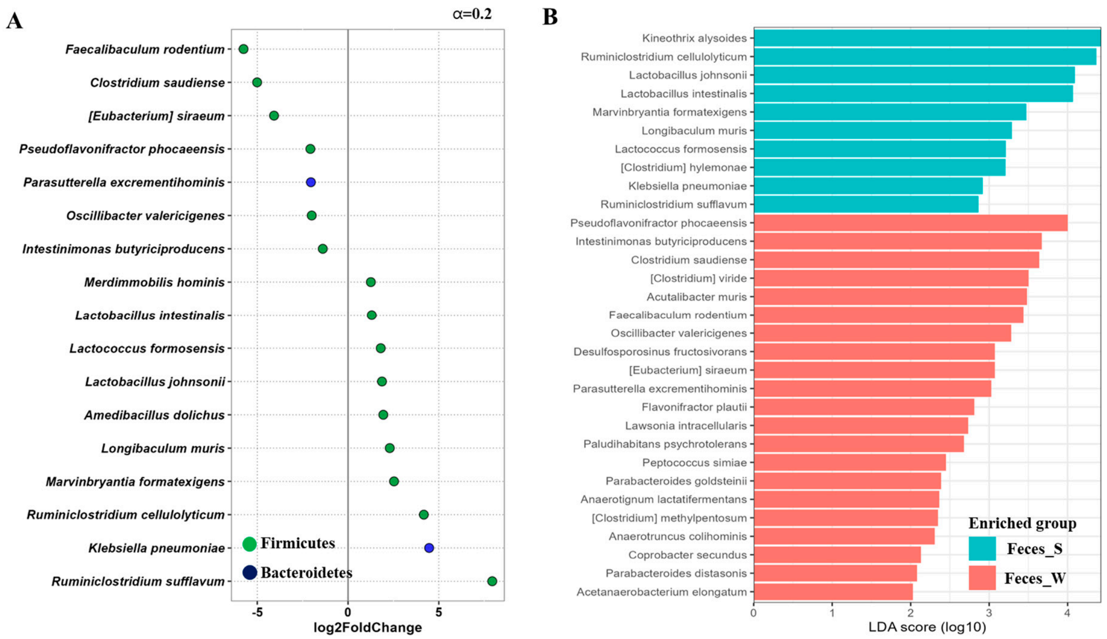

Figure S4. Differential analysis of GI gut microbiota in wild-type (W) and severe lupus (S) MRL/lpr mice . (A) Volcano plot showing differentially abundant GI microbial species between severe lupus (GI_S) and wild-type (GI_W) mice, as determined by DESeq analysis using read counts. Species from the phylum *Fir micutes* are shown in green, while species from *Bacteroidetes* are shown in blue. Positive values indicate s pecies enriched in severe lupus mice, while negative values indicate those enriched in wild-type mice. (B) Linear discriminant analysis (LDA)effect size (LEfSe) plot identifying differentially enriched GI microbial species between severe lupus (GI_S) and wild-type (GI_W) mice based on read counts. Species enriched i n the severe group are shown in blue, while those enriched in the wild-type group are shown in red. High er LDA scores indicate a greater contribution to the respective group’s microbial composition.

Table S1. Differentially abundant fecal and GI gut microbial species based on read count in mild and seve re lupus MRL/lpr mice.

Sample	Strain	Abundance based on read count (%)		
		Lupus-weak	Lupus-medium	Lupus-strong
Fecal	*Clostridium saudiense*	0.00	0.67	0.62
	Turicibacter sanguinis	0.06	3.78	3.86
	Ligilactobacillus animalis	0.19	0.42	0.56
	Hoylesella oralis	1.39	0.73	0.58
	Phocaeicola sartorii	2.14	0.83	0.56
	Eisenbergiella massiliensis	1.40	0.47	0.21
GI	*Clostridium saudiense*	0.01	0.42	0.56
	Pseudoflavonifractor phocaeensis	0.27	1.04	1.38
	Intestinimonas butyriciproducens	0.20	0.48	1.73
	Ruminiclostridium cellulolyticum	2.29	0.28	0.20
	Lactobacillus johnsonii	1.64	0.58	0.38
	Kineothrix alysoides	11.75	9.71	6.97
